# Locally structured correlation (LSC) plots describe inhomogeneity in normally distributed correlated bivariate variables

**DOI:** 10.1186/s13690-021-00748-4

**Published:** 2022-01-17

**Authors:** Rebekka Mumm, Christiane Scheffler, Michael Hermanussen

**Affiliations:** 1 University of Potsdam, Institute of Biochemistry and Biology, Human Biology, Potsdam, Germany; 2 Aschauhof, 24340 Altenhof, Germany

**Keywords:** Standard deviation, Locally structured standard deviation, Locally structured correlation, Variance

## Abstract

**Background:**

The association between bivariate variables may not necessarily be homogeneous throughout the whole range of the variables. We present a new technique to describe inhomogeneity in the association of bivariate variables.

**Methods:**

We consider the correlation of two normally distributed random variables. The 45° diagonal through the origin of coordinates represents the line on which all points would lie if the two variables completely agreed. If the two variables do not completely agree, the points will scatter on both sides of the diagonal and form a cloud. In case of a high association between the variables, the band width of this cloud will be narrow, in case of a low association, the band width will be wide. The band width directly relates to the magnitude of the correlation coefficient. We then determine the Euclidean distances between the diagonal and each point of the bivariate correlation, and rotate the coordinate system clockwise by 45°. The standard deviation of all Euclidean distances, named *“global standard deviation”*, reflects the band width of all points along the former diagonal. Calculating moving averages of the standard deviation along the former diagonal results in *“locally structured standard deviations”* and reflect patterns of *“locally structured correlations (LSC)”.* LSC highlight inhomogeneity of bivariate correlations. We exemplify this technique by analyzing the association between body mass index (BMI) and hip circumference (HC) in 6313 healthy East German adults aged 18 to 70 years.

**Results:**

The correlation between BMI and HC in healthy adults is not homogeneous. LSC is able to identify regions where the predictive power of the bivariate correlation between BMI and HC increases or decreases, and highlights in our example that slim people have a higher association between BMI and HC than obese people.

**Conclusion:**

Locally structured correlations (LSC) identify regions of higher or lower than average correlation between two normally distributed variables.

## Background

The association between bivariate variables may not necessarily be homogeneous throughout the whole range of the variables so that the independent variable predicts the criterion variable with different predictive power along the abscissa. This is an old problem, relevant for anthropology, public health, and many other disciplines.

We give a public health example: Slim people have both low body mass index (body weight divided by height square, BMI) and narrow hip circumference (HC). Obese people have both high BMI and a much wider HC. Yet, the strength of this correlation varies along the X- and the Y-axis. The correlation appears much better in the slim than in the obese people. We say, the correlation is not homogeneous. Yet, approaching this phenomenon statistically is difficult. When we restrict the range of either one of the variables (i.e. when we either look at only the slim, or only the obese) to better focus on either one group, the resulting correlation coefficient within the restricted range will be reduced [1].

We present a new technique to better describe inhomogeneity in the association of bivariate variables.

## Sample

We exemplify the new technique by analyzing data on body mass index (BMI) and hip circumference (HC) in 6313 healthy adults aged 18 to 70 years from former East Germany. Information on height, weight, age, sex and several other anthropometric variables e.g. hip and waist circumference, sitting height, etc. are available. Details of these data and available variables were published elsewhere [2]. Height, weight and HC had been measured following standard procedure [3]. BMI was calculated in the usual way (kg/m^2^). Sex-specific z-scores for BMI and HC were calculated:

$$ z- score=\frac{measured\ value\ of\ individual- mean\ of\ group}{standard\ deviation\ (SD)\  of\ group} $$

## Methods

Consider X and Y two normally distributed random variables with *X*~*N*(0, 1) and *Y*~*N*(0, 1), $\overline{x}$ and $\overline{y}$ the arithmetic mean and ${s_x}^2$ and ${s_y}^2$ the corrected sample variance. If variables *X* and *Y* do not have standard normal distribution (e.g. this is the case for BMI and HC in our example of 6,313 healthy adults), z-transformation has to be used first for both variables. The Pearson correlation *r* between *X* and *Y* can be written as

1 $$ {\displaystyle \begin{array}{c}r= Cor\left(X,Y\right)\\ {}=\frac{\sum_{i=1}^n\left({x}_i-\overline{x}\right)\left({y}_i-\overline{y}\right)}{\sqrt{\sum_{i=1}^n{\left({x}_i-\overline{x}\right)}^2{\left({y}_i-\overline{y}\right)}^2}}\\ {}\begin{array}{c}=\frac{\sum_{i=1}^n\left({x}_i-\overline{x}\right)\bullet \left({y}_i-\overline{y}\right)}{\sqrt{{s_x}^2\bullet \left(n-1\right)\bullet {s_y}^2\bullet \left(n-1\right)}}\\ {}\overset{{s_x}^2={s_y}^2=1}{\overbrace{=}\ }\frac{\sum_{i=1}^n\left({x}_i-\overline{x}\right)\bullet \left({y}_i-\overline{y}\right)}{\sqrt{{\left(n-1\right)}^2}}\\ {}\overset{\overline{x}=\overline{y}=0}{\overbrace{=}\ }\ \frac{1}{n-1}\ {\sum}_{i=1}^n{x}_i\bullet {y}_i\end{array}\end{array}} $$

If the two variables completely agreed (which is not the case in measures of BMI and HC), all measurements would lie on the 45° diagonal through the origin of the coordinates. If the two variables do not completely agree (as this is the case in measures of BMI and HC), the measurement points scatter on both sides of the diagonal forming a cloud of measurements. In case of a high association between the variables, the band width of this cloud will be narrow, in case of a low association, the band width will be wide. The band width directly relates to the magnitude of the correlation coefficient. We discuss the case that the two variables do not completely agree, i.e. *r* > 0. The diagonal can be written as $g:\overrightarrow{x}=\overrightarrow{a}+t\bullet \overrightarrow{b},t\ \epsilon\ \mathbb{R}$ with $$ \overrightarrow{a}=\left(\begin{array}{c}0\\ {}0\end{array}\right),\overrightarrow{b}=\left(\begin{array}{c}1\\ {}1\end{array}\right) $$. Therefore

2 $$ g:\overrightarrow{x}=\left(\begin{array}{c}0\\ {}0\end{array}\right)+t\bullet \left(\begin{array}{c}1\\ {}1\end{array}\right),t\ \epsilon\ \mathbb{R} $$

We determine the Euclidean distance *d* between the diagonal *g* and any point *P*(*x*_*i*_| *y*_*i*_) *ϵ ℝ*^2^ with *x*_*i*_ *ϵ X* and *y*_*i*_ *ϵ Y*. With Eq. (2) we get

3 $$ d\left(g,P\right)=\frac{\left|\left(\begin{array}{c}1\\ {}1\end{array}\right)\times \left(\left(\begin{array}{c}{x}_i\\ {}{y}_i\end{array}\right)-\left(\begin{array}{c}0\\ {}0\end{array}\right)\right)\right|}{\left|\left(\begin{array}{c}1\\ {}1\end{array}\right)\right|}=\frac{\left|{y}_i-{x}_i\right|}{\sqrt{2}} $$

We calculated the mean squared error (MSE) as the sum of the distance *d* for all random points *P*(*x*_*i*_| *y*_*i*_) *ϵ ℝ*^2^ with *x*_*i*_ *ϵ X* and *y*_*i*_ *ϵ Y*.

4 $$ {\displaystyle \begin{array}{c} MSE=\frac{1}{n-1}{\sum}_{i=1}^n{\left(\frac{\left|{y}_i-{x}_i\right|}{\sqrt{2}}\right)}^2\\ {}=\frac{1}{n-1}{\sum}_{i=1}^n\frac{{y_i}^2-2{x}_i{y}_i+{x_i}^2}{{\left(\sqrt{2}\right)}^2}\\ {}=\frac{1}{2\left(n-1\right)}{\sum}_{i=1}^n{y_i}^2-2{x}_i{y}_i+{x_i}^2\\ {}=\frac{1}{2\left(n-1\right)}{\sum}_{i=1}^n{y_i}^2-2\bullet {\sum}_{i=1}^n{x}_i{y}_i+{\sum}_{i=1}^n{x_i}^2\\ {}=\frac{1}{2}\left(\underset{=1}{\underbrace{\frac{1}{n-1}{\sum}_{i=1}^n{y_i}^2}}-\frac{2}{n-1}{\sum}_{i=1}^n{x}_i{y}_i+\underset{=1}{\underbrace{\frac{1}{n-1}{\sum}_{i=1}^n{x_i}^2}}\right)\\ {}\overset{{s_x}^2={s_y}^2=1}{\overbrace{=}\ }\frac{1}{2}\left(1-\frac{2}{n-1}{\sum}_{i=1}^n{x}_i{y}_i+1\right)\\ {}=\frac{1}{2}\ \left(2-\frac{2}{n-1}{\sum}_{i=1}^n{x}_i{y}_i\right)\\ {}\overset{(1)}{\overbrace{=}}1-\underset{=r}{\underbrace{\frac{1}{n-1}{\sum}_{i=1}^n{x}_i{y}_i}}\\ {}=1-r\end{array}} $$

The observed (corrected) dispersion ${\hat{s}}^2$, or band width of the cloud of points alongside of the diagonal is equivalent to what is commonly considered the variance. With (4), the observed (corrected) dispersion ${\hat{s}}^2$ can be described by

5 $$ {\hat{s}}^2=1-r,\mathrm{for}\ r>0. $$

Similar for *r* < 0 the dispersion ${\hat{s}}^2$ can be described using the same arguments as

6 $$ {\hat{s}}^2=1+r,\mathrm{for}\ r<0. $$

With (5) and (6) we get for each *r ϵ ℝ*, *r* ≠ 0

7 $$ {\displaystyle \begin{array}{c}{\hat{s}}^2=\left\{\begin{array}{c}1+r,r<0\\ {}1-r,r>0\end{array}\right.\\ {}=1-\left|r\right|\end{array}} $$

In summary, the band width of the scattered points along both sides of the 45° diagonal is directly related to the bivariate Pearson correlation coefficient (Fig. 1.1).

**Fig. 1:**
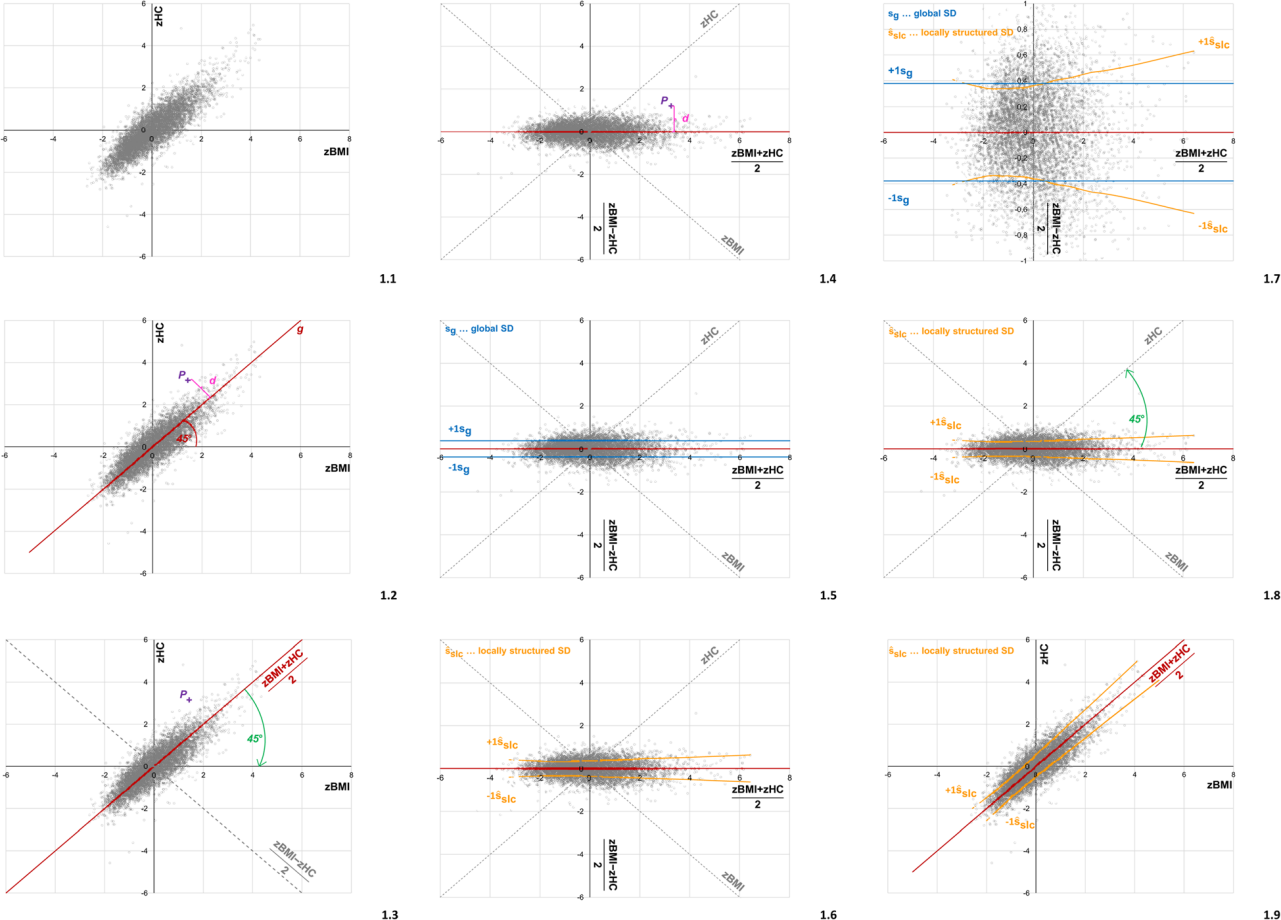
Locally structured standard deviation ${\hat{s}}_{lsc}$, and locally structured correlation ${\hat{r}}_{lsc}$ between BMI and HC (details provided in the text)

The observed dispersion ${\hat{s}}^2$ of a sample derived from the Euclidean distances between the diagonal *g* and each point (Eqs. 3 and 7), also called *“global variance”* ${s}_g^2$ reflects the average scattering of all points along the diagonal of the original correlation plot, i.e. the *“global correlation” r*_*g*_ as given by Pearson correlation between *X* and *Y* and the *“global standard deviation” s*_*g*_

$$ {s}_g^2=1-{r}_g $$

In order to identify inhomogeneity in the band width of all points along the former diagonal and therefore to describe the magnitude of the association separately for the slim, for the normal, and for the obese, we compute *“locally structured standard deviations”* ${\hat{s}}_{lsc}$ by the following two steps. First, we calculate moving averages [5] of standard deviations to better describe the local magnitude of scattering along the new abscissa. In a second step, the moving averages are smoothed e.g. by using LOESS (Locally Estimated Scatterplot Smoothing) technique [6]. Other smoothing techniques e.g. LOWESS (Locally Weighted Scatterplot Smoothing) or GAM (Generalized Additive Modelling) might be applicable as well.

The resulting smoothed moving averages of the standard deviation is now called *“locally structured standard deviations”* ${\hat{s}}_{lsc}$. The pattern of *“locally structured standard deviations”* ${\hat{s}}_{lsc}$ reflects the *“locally structured correlations”* ${\hat{r}}_{lsc}$, written as

8 $$ {\hat{r}}_{lsc}=1-{{\hat{s}}_{lsc}}^2 $$

*“Locally structured correlations”* depict the “local” association between BMI and HC within the full range from slim to normal, and to obese persons.

For all analyses the statistical software R [4] was used.

## Results

We exemplify this approach. Figure 1.1 and 1.2 depict the correlation plot for BMI z-scores (zBMI) and HC z-scores (zHC) in 6313 healthy East German adults. The correlation is not homogeneous, though this may not be immediately visible.

For visualization, we rotate the coordinate system clockwise by 45° (Fig. 1.3 and 1.4) and define two new Cartesian axes. The former diagonal line has now turned into the new abscissa described by $\frac{zBMI+ zHC}{2}$. The new ordinate is given by $\frac{zBMI- zHC}{2}$. The *“global standard deviations” s*_*g*_ between zBMI and zHC of slim, normal and obese people combined are presented as horizontal lines in Fig. 1.5. Figure 1.6 illustrates the *“locally structured standard deviations”* ${\hat{s}}_{lsc}$ of zBMI and zHC after smoothing the moving averages of the standard deviation (=local pattern of scattering). In our example, the standard technique for scatterplot smoothing LOESS was used. Figure 1.7 magnifies the pattern of ${\hat{s}}_{lsc}$. Rotating the coordinate system back by 45° (counter-clockwise) (Fig. 1.8 and 1.9) shows the *locally structured correlations (LSC) plot* and highlights the inhomogeneity of the bivariate correlation between BMI and HC. LSC-plots identify regions within correlation plots where the predictive power increases or decreases (increasing or decreasing “*locally structured standard deviations”* ${\hat{s}}_{lsc}$*)*. Predictive power is highest around BMI and HC z-values between -1 and 0 (low locally structured standard deviations ${\hat{\mathrm{s}}}_{\mathrm{lsc}}$) and decreases with increasing BMI and/or HC (high locally structured standard deviations ${\hat{\mathrm{s}}}_{\mathrm{lsc}}$ ).

**Fig. 2:**
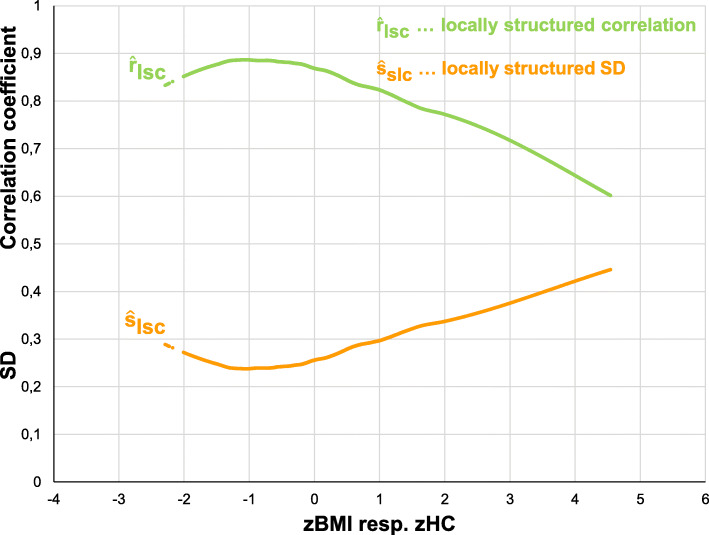
Reciprocal dynamics of the locally structured standard deviation ${\hat{s}}_{lsc}$ and the locally structured correlation ${\hat{r}}_{lsc}$ indicating regions of higher or lower than average correlation between BMI and HC

Figure 2 illustrates the reciprocal dynamics of locally structured ${\hat{s}}_{lsc}$ and the locally structured correlation ${\hat{r}}_{lsc}$. Locally structured correlations ${\hat{r}}_{lsc}$ indicate regions of higher or lower than average (global) correlation between BMI and HC. Correlation is highest for zBMI- or zHC-values of -1. The correlation decreases for z-values above 0 indicating an increase of variability in zBMI and zHC and thereby demonstrates a weaker association than average between the two variables. I.e. slim people have a higher association between BMI and HC than obese people.

Negative associations of bivariate variables that are also common in public health can be analyzed in analogy.

## Discussion

In public health the interaction between bivariate variables is often described by using the Pearson correlation between these variables. The Pearson correlation assumes homogenous linear relationships but this may not necessarily be the case as we have seen in the example of BMI and HC. Localizing particular areas within which the strength of the interaction between two bivariate variables may be greater or smaller, is not trivial, and also estimating the magnitude of this phenomenon is difficult. Restricting the range of one of the variables (e.g. assessing the association between BMI and HC only in the slim or only the obese) is not an appropriate statistical method.

Restrictions of range reduce the power of an experiment, because correlations are attenuated by reduced variability, a problem well known. The problem of restricted ranges in correlation was first exemplified in the classical study by Thorndike in 1949 [7]. Bland and Altman [1] further discussed this issue and defined restricted ranges of variables on the x-axis but state that a detailed analysis of subgroups within a variable is not appropriate and refer to using regression instead of correlation. Several additional methods exist for correcting correlations for range restriction [8]. Wiberg and Sundström [8] tested two approaches for correcting restricted correlations, but concluded that further studies are necessary – a final solution for the problem is still unknown but widely needed for public health.

Figure 2 illustrates the inhomogeneity of the correlation between zBMI and zHC in our example. In slim people the correlation between zBMI and zHC with *r* close to 0.9, has a high predictive power, indicating that adults with low BMI are very likely to also have a low HC. The pattern of “*locally structured correlation”* illustrates the magnitude at which the association between BMI and HC depends on the body’s fat depots.

Many variables such as physical activity, child growth, adult height, income, the association of socio-economic status and risk of infectious diseases etc. are highly interrelated and usually far from being homogenous [9, 10].

## Conclusion

In public health, complex interactions between social, economic and anthropological variables are common. Inhomogeneity in the association of these variables may further jeopardize the understanding of these interactions.

We present a new method for studying local variability within correlation plots of bivariate variables by creating locally structured correlations (LSC). LSC identify regions of higher or lower than average association within the correlation of two normally distributed variables. In contrast to existing statistical methods, locally structured correlations are not based on range restriction, but directly analyze local inhomogeneity along the 45° diagonal axis crossing the origin of coordinates. The new method helps to improve the understanding of complex interactions of variables in public health studies.

## Data Availability

Not applicable.

## Abbreviations

BMI: Body mass index
HC: Hip circumference
LSC: Locally structured correlation
zBMI: z-scores for BMI
zHC: z-scores for hip circumference
SD: standard deviation
